# Beyond the Inhibition of Return of Attention: Reduced Habituation to Threatening Faces in Schizophrenia

**DOI:** 10.3389/fpsyt.2014.00007

**Published:** 2014-01-29

**Authors:** Frank K. Hu, Shuchang He, Zhiwei Fan, Juan Lupiáñez

**Affiliations:** ^1^Department of Psychology, University of Maryland, College Park, MD, USA; ^2^Department of Psychology, Peking University, Beijing, China; ^3^Department of Experimental Psychology, University of Granada, Granada, Spain; ^4^Mind, Brain and Behavior Research Center, University of Granada, Granada, Spain

**Keywords:** schizophrenia, inhibition of return, disengagement, habituation/detection cost

## Abstract

Attention deficits are prominent among the core symptoms of schizophrenia. A recent meta-analysis has suggested that patients with schizophrenia have a deficit in endogenous disengagement of attention. In this research, we used a standard spatial cueing paradigm to examine whether the attention deficit of such patients is due to impaired attentional disengagement or defective novelty detection/habituation processes. In a spatial cueing procedure with peripheral non-predictive cues and a detection task, we manipulated the valence of either the cue or the target (i.e., a threatening vs. scrambled face) in two separate experiments. The control group exhibited a smaller inhibition of return (IOR) effect only when the target had an emotional load, not when the cue had an emotional load. In the patient group, a larger emotional effect appeared when the threatening face was the target; by contrast, no effect of valence was observed when the threatening face was the cue: IOR was delayed or completely absent independently of valence. The present findings are in conflict with the hypothesis that IOR is due to the disengagement of attention and the subsequent inhibition to return. Instead, they seem to suggest a cost in detecting new information at a previously cued location. From this perspective, it seems that patients with schizophrenia might have a deficit in detecting new information and considering it as new in the current context.

## Introduction

Multi-dimensional cognitive impairment has been consistently reported in patients with schizophrenia (1–4). Such patients have been found to have deficits in a broad range of cognitive tasks that are considered to involve inhibitory attentional processes, such as priming, covert orienting, latent inhibition, antisaccade, sensorimotor gating, and executive tasks [e.g., Ref. (5–10)]. In line with this, there is evidence that people with schizophrenia perform poorly on attention tasks that require vigilance and quick responses [e.g., Ref. (11, 12)].

A phenomenon that seems to be well suited for studying attention deficits in schizophrenia is “inhibition of return” (IOR) (13–15), which we shall explain next. In a typical experiment, participants perform a simple detection task, pushing a response button upon detection of the appearance of a small dot (i.e., the target), which can appear in one of two boxes (one on each side of the fixation point) on a computer screen in front of them. Before the target is presented, an exogenous cue (i.e., the brightening of one of the boxes) marking any of the possible target locations is used to capture attention. When the cue–target interval [i.e., stimulus onset asynchrony (SOA)] is less than about 250 ms, responses are usually faster when the visual targets appear at the cued location. At longer SOAs, however, this facilitation effect becomes an opposite effect and responses are slower at cued locations than at uncued locations [see Chica et al. (16), for a review of results typically observed with this procedure]. This inhibitory effect was first reported by Posner and Cohen (15) and later named “inhibition of return,” IOR (17). In some studies, a second cue (usually called “cue-back”) is presented at fixation between the initial cue and the final target with the purpose of disengaging attention from the initially cued location. Recently, Mushquash et al. (18) summarized the literature exploring attention deficits in schizophrenia with IOR procedures. Using a meta-analytic approach, the authors reported that patients with schizophrenia showed a delayed or reduced IOR effect in single-cue procedures whereas their IOR pattern was more consistent with that of control participants when cue-back procedures were used instead. Results were interpreted as indications of a deficit in endogenous disengagement of attention in schizophrenia. According to the authors, the smaller or delayed IOR “may not be evidence for a deficit in exogenous control of attention, but instead, a deficit in endogenous or voluntary control of attention” (p. 56).

This analysis is consistent with the traditional view of IOR (15). Posner et al. originally considered that the IOR effect was due to a mechanism that prevents reorienting attention to previously attended locations. When attention is withdrawn (i.e., disengaged from the cued location), it is inhibited from moving back and performance is impaired at the cued location. In short, this view assumes that IOR is a consequence of attentional disengagement and that there would be no IOR if attention was not disengaged from the cued location. In fact, the cue-back procedure has been used in a few studies on IOR in order to enhance disengagement of attention (19). The conclusions reached by Mushquash et al. as well as other empirical findings [e.g., Ref. (19–21)] are clearly in line with this attention disengagement hypothesis.

However, recent evidence indicates that attention disengagement from the cued location is neither necessary nor sufficient for the IOR effect to be observed [e.g., Ref. (22)]. It has been shown that IOR can be observed at locations where attention is not disengaged from the cued location (23–25). Hence, disengagement of attention is not necessary for IOR to be observed. What is more, it has also been shown that, under some circumstances, facilitation instead of IOR is observed even after attention is disengaged from the cued location (26, 27). Thus, disengagement of attention is not sufficient for IOR to be observed. Consequently, researchers are proposing alternative models to account for the dissociation between IOR and attentional disengagement. For example, Dukewich (28) recently reconceptualized IOR as the result of the habituation of the orienting response instead of the result of attentional disengagement. According to this view, the presence of a similar preceding event (i.e., cue) leads to a weakened orienting response to the later event (i.e., target) [e.g., Ref. (29–31)]. Similarly, Lupiáñez et al. (22, 32, 33) proposed that the IOR effect in fact reflects a cost in rapidly detecting the appearance of new objects or events that are similar to those that have captured attention before. By treating the target as an update of the cue [i.e., an update of the object-file representation of the cue; (34)], cueing a location hinders detection of a subsequent event at the very same location (30, 31). Importantly, these new models question whether the abnormality in visual attention observed in schizophrenia is due to a deficit in attentional disengagement, as suggested by Mushquash et al.

The present research was designed to determine whether the attention deficit in schizophrenia is due to impaired attentional disengagement or defective novelty detection/habituation processes. Two groups of participants (i.e., schizophrenia group and control group) were recruited. In order to study habituation of attentional capture and disengagement of attention, we used emotional stimuli (i.e., threatening faces), following a few IOR studies in which emotional stimuli were used either as cues or targets [e.g., Ref. (35–40)]. We used threatening faces as stimuli because it has been observed that people show enhanced attentional orienting and engagement to such stimuli (41) while disengagement of attention from such emotional stimuli tends to be delayed (40, 42). Similarly, there is literature that may be interpreted as evidence that threatening facial expressions have a special status in capturing visual attention [e.g., Ref. (43–46)]. In fact, perception of threatening faces is the most developed visual perceptual skill in human beings (45, 47). As regards patients with schizophrenia, recent evidence indicates that they have both attentional and emotional dysfunctions (11, 48, 49). For instance, Strauss et al. (50, 51) reported that people with schizophrenia had difficulties disengaging attention from unpleasant stimuli. Moreover, a recent review has provided consistent evidence of increased vigilance and selective attention toward negative facial expressions in individuals with major depression (52). Therefore, we considered that it would be comparatively easier for patients with schizophrenia to orient to and be engaged by threatening stimuli but more difficult to disengage attention from those emotional stimuli.

In contrast with previous studies in which the role of emotionality in IOR was explored using localization or discrimination tasks, we used a simple detection task in our two experiments. This allowed us to verify whether emotionality plays a stronger role in IOR in patients with schizophrenia when emotionality itself is completely irrelevant to the task at hand. In Experiment 1, the cue was either a threatening face or its corresponding scrambled face and the target was a square. In Experiment 2, the cue was a square and the target was either a threatening face or its corresponding scrambled face. According to the attention disengagement view of IOR, less or delayed IOR should be expected if the cue involves threatening information, as it will be more difficult to disengage attention from such information (38, 50). To the extent that patients with schizophrenia have problems with endogenous disengagement of attention, they will show a further reduction or delay in IOR with threatening cues as compared to control participants. By contrast, this theory predicts no emotional effect if only the target valence is manipulated, as everything is the same for all neutral and emotional target trials before the target is presented. In contrast to the attention disengagement view of IOR, the habituation or detection cost theory of IOR predicts a stronger effect on IOR when the target has an emotional valence than when the cue has such valence. More specifically, it predicts a reduced or delayed IOR effect for threatening targets, as those stimuli will be less affected by habituation than neutral targets (41). To the extent that patients with schizophrenia will be particularly attracted by negative stimuli as compared to control participants (52), they will show more reduced IOR (i.e., reduced habituation to salient stimuli) for such negative targets. This distinction between deficits in attentional disengagement and reduced habituation processes will have important theoretical implications regarding the characterization of attentional deficits in schizophrenia.

## Experiment 1

In Experiment 1, we tested the effect of negative cue valence (i.e., threatening facial expression) on IOR in patients with schizophrenia and control participants. The target was a peripherally presented small filled-in square that was preceded by a peripheral non-predictive cue (i.e., either a threatening face or a corresponding scrambled face, a neutral cue). Participants were required to push a button once they detected the square target. Our aim was to determine whether and how the IOR would be modulated by the valence of the cue (i.e., threatening face vs. scrambled face) in the two groups. According to the disengagement view of IOR, we should find reduced IOR for threatening cues, especially in the patient group. By contrast, according to the habituation or detection cost theory of IOR, we should find no effect of emotionality of the cue on the size of the IOR effect. Moreover, if patients with schizophrenia have an impairment in novelty detection ability, which is the basis of the IOR effect, they should show a reduced IOR effect regardless of the emotionality of the cue.

### Participants

Our sample included 20 individuals meeting the criteria for schizophrenia of the Diagnostic and Statistical Manual of Mental Disorders (DSM-IV) (53) and 15 healthy controls (see Table 1). The patient group was composed of inpatients from Beijing Psychiatric Hospital, Beijing, China. Patients’ diagnoses were confirmed with structured clinical interviews that were in accordance with the DSM-IV. Each patient was evaluated by at least two experienced psychiatrists at the hospital. Before conducting the experiments, patients were carefully screened to rule out any potential disorders that might alter brain functioning (e.g., mental retardation). Participants were excluded if they met any of the following conditions: (1) substance abuse or dependence during the 6 months immediately prior to the study; (2) a history of head injury with documented sustained loss of consciousness, neurological sequelae, or both; or (3) abnormal cerebral metabolism arising from neurological illness or any other disorder. In the control group, all participants were undergraduate or graduate students recruited from Peking University, Beijing. All participants reported normal or correct-to-normal vision and being right-handed and were naïve as to the purpose of the experiment. Since all patients were clinically stable, most had undergone long-term treatment and were older than the comparison group. All participants gave their oral and written informed consent. The study was conducted in accordance with the Declaration of Helsinki and was approved by the local ethics committee.

**Table 1:** **Characteristics of the groups studied in Experiment 1 (patients with schizophrenia and control participants)**.

Participants’ characteristics	Schizophrenia (*n* = 20)	Healthy controls (*n* = 15)	*t* Value	*p* Value
Age (years)	34.0 (1.64)	22.8 (0.611)	5.67	0.001
Gender (female ratio)	20/20	9/15	3.06	0.009
Education level (years)	9.30 (0.603)	13.8 (0.296)	6.05	0.001
Right-handedness	20/20	15/15		
Length of illness (years)	11.55 (1.86)			

*Standard error (SE) is shown in brackets for age, education level, and length of illness*.

### Apparatus and procedure

**Figure 1:**
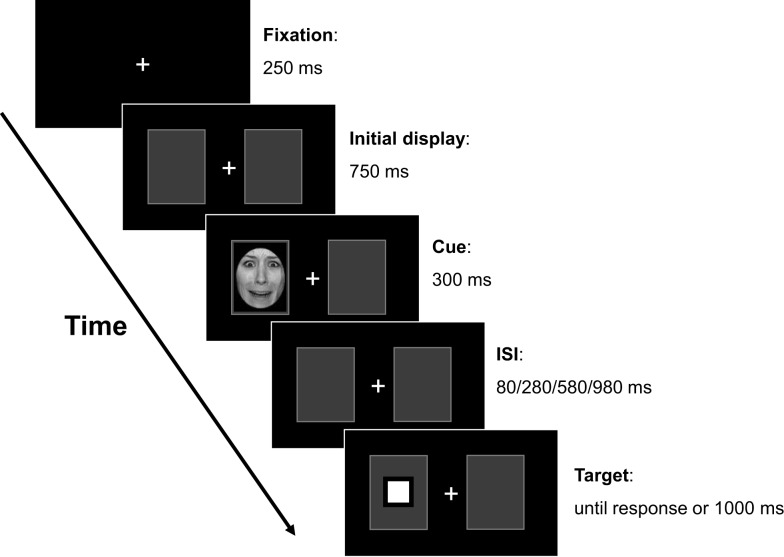
**Schematic of procedure used in the spatial cueing task**. Each trial had the following sequence: fixation, initial display, cue, inter stimulus interval (ISI), target, and response. The picture shows an example of a cued trial with a threatening face target (not drawn exactly to scale).

The experiment was run in dimly-lit rooms. Participants were seated approximately 65 cm away from the monitor. A computer keyboard was directly in front of the participant and the “B” key was used as the response device. The experimental procedure was as follows: each trial began with a fixation display (see Figure 1) that remained on the screen for 250 ms, followed by a 750 ms initial display, in which one box was added on each side of the fixation (visual angle: 5.60° × 5.60°). The distance between the center of the peripheral box and fixation was 8.40°. The third display was a critical cue whose valence (i.e., threatening face vs. scrambled face, luminance matched) was manipulated and whose size was adjusted to the box frame. The cue was presented for 300 ms. After a time variable interval (80, 280, 580, or 980 ms), the target display appeared and participants were required to hit the response key as soon as possible once they detected the target (i.e., a square with a 0.90° × 0.90° visual angle). Stimuli were presented with E-Prime software (54). Each participant completed 20 practice trials followed by four runs of 71 trials. Each run included seven catch trials in which no target appeared and participants were required to withhold their responses. The total of 256 randomized trials were divided equally into 16 trials for each combination of SOA (380, 580, 880, and 1280 ms), Cue Valence (threatening face vs. scrambled face), and Cueing (valid vs. invalid). All levels of the three variables were mixed within each run.

### Results and discussion

**Figure 2:**
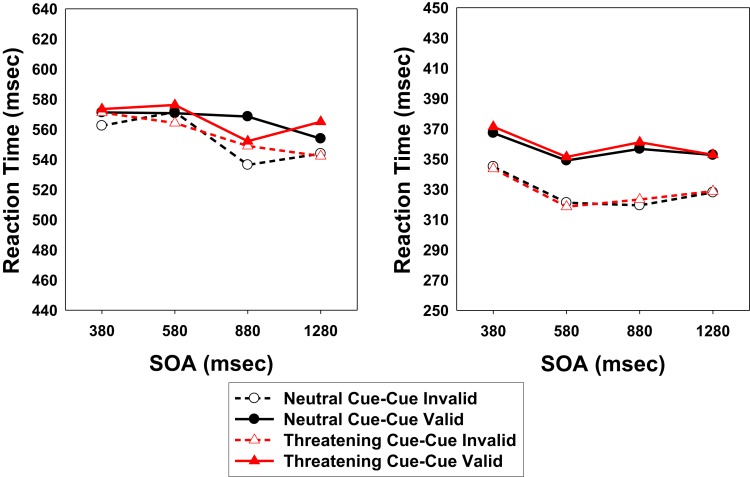
**Target detection times of Experiment 1, broken down by Cueing (cued vs. uncued location), Cue Valence (threatening vs. neutral), and Stimulus Onset Asynchrony (SOA)**. Left panel: patient group; right panel: control group.

Reaction times (RTs) above and below ±2.5 SD from the condition-specific mean were eliminated from the data analyses. Less than 5% of the trials were discarded following this criterion (controls: 3.8%; patients: 4.6%). Figure 2 shows the mean target detection times, broken down by SOA, Cueing, and Cue Valence. Table 2 shows mean RTs and percentage of errors (misses). Mean RTs were subjected to a 2 × 4 × 2 × 2 mixed analysis of variance (ANOVA) with group (patients vs. controls) as the between-participants factor and SOA (380, 580, 880, and 1280 ms), Cue Valence (threatening face vs. scrambled face), and Cueing (valid vs. invalid) as within-participants factors. From that stage, the Greenhouse–Geisser correction was used when necessary to mitigate violations of the sphericity assumption (55). The analysis revealed a main effect of SOA, *F*(2, 72) = 8.25, *p* < 0.001, a main effect of Group, *F*(1, 33) = 41.26, *p* < 0.001, and an SOA × Group interaction, *F*(3, 99) = 4.47, *p* = 0.005. In the control group, RTs decreased from the first to the second SOA and then remained similar for the remaining SOAs. By contrast, in the patient group, response speed only increased at the third and fourth SOAs as compared to the second SOA level.

**Table 2:** **Mean reaction times (ms) and. accuracy as a function of Cue Valence (threatening vs. neutral), Cueing (valid vs. invalid), and SOA in Experiment 1**.

**EXPERIMENT 1**								
	SOA							
	380		580		880		1280	
	Cue_invalid	Cue_valid	Cue_invalid	Cue_valid	Cue_invalid	Cue_valid	Cue_invalid	Cue_valid
**RT (ms)**
Control neutral cue	344.98 (11.66)	367.20 (10.24)	321.09 (13.71)	349.00 (11.10)	319.37 (12.28)	356.71 (13.90)	327.91 (11.37)	352.68 (13.01)
Control threatening cue	343.60 (11.23)	371.33 (12.37)	318.48 (10.46)	351.36 (11.95)	323.24 (12.53)	361.06 (12.55)	328.75 (13.29)	352.79 (12.57)
Patient neutral cue	562.40 (30.95)	571.25 (26.12)	571.54 (31.35)	570.70 (27.58)	536.40 (29.67)	568.49 (29.88)	543.86 (30.78)	553.85 (29.67)
Patient threatening cue	571.11 (29.34)	573.47 (26.80)	564.21 (29.67)	576.16 (26.44)	548.97 (30.98)	552.14 (26.59)	542.25 (29.11)	565.05 (28.08)
**Accuracy**
Control neutral cue	0.99 (0.01)	1.00 (0.00)	0.99 (0.01)	0.99 (0.01)	1.00 (0.00)	1.00 (0.00)	1.00 (0.00)	1.00 (0.00)
Control threatening cue	1.00 (0.00)	0.99 (0.01)	0.99 (0.01)	1.00 (0.00)	1.00 (0.00)	1.00 (0.00)	1.00 (0.00)	1.00 (0.00)
Patient neutral cue	0.93 (0.03)	0.89 (0.03)	0.91 (0.02)	0.93 (0.02)	0.91 (0.03)	0.92 (0.02)	0.92 (0.02)	0.91 (0.03)
Patient threatening cue	0.89 (0.03)	0.88 (0.04)	0.93 (0.02)	0.93 (0.02)	0.94 (0.02)	0.93 (0.03)	0.93 (0.02)	0.94 (0.02)

*Standard error (SE) is shown in brackets*.

More importantly, we observed a main effect of Cueing, *F*(1, 33) = 18.59, *p* < 0.001, that differed between groups, confirming our predictions. Although the Cueing × Group interaction was only marginally significant, *F*(1, 33) = 3.67, *p* = 0.064^1^, a further analysis indicated that only the control group showed a significant IOR effect, *F*(1, 14) = 62.41; *p* < 0.001. By contrast, and supporting our predictions, the IOR effect shown by patients was much smaller in size and not significant, *F*(1, 19) = 2.18, *p* = 0.156. It should be noted that the main effect of Cue Valence was not significant, *F*(1, 33) = 0.80, *p* = 0.377. No other significant effects were found. Complete lists of the results from the statistical tests are presented in the Appendix. Finally, the analysis of errors (i.e., missed responses) only revealed a main effect of Group, indicating that patients missed the target (8.20%) more frequently than controls (0.36%), *F*(1, 33) = 11.83, *p* = 0.002.

In Experiment 1, we observed a clear data pattern when cue valence was manipulated. In the control group, we found a similar significant IOR effect for both neutral and emotional (i.e., threatening) cues. In the patient group, however, IOR was completely absent or delayed, regardless of the emotionality of the cue. It is worth noting that patients showed reduced or no IOR as compared to control participants, replicating previous results [e.g., Ref. (56)]. According to the attention disengagement view, a reduced IOR effect should be expected for threatening cues since participants would find it more difficult to disengage from emotionally relevant cues [e.g., Ref. (38, 50)]. However, this was not the case in our study. Yet, the present finding is in line with the habituation/detection cost hypothesis of IOR, according to which patients with schizophrenia have a deficit in detecting new information. This is discussed further in the Section “General Discussion” below.

However, it could be argued that patients’ major deficit in attentional disengagement led them not to exhibit any IOR even for neutral cues (i.e., a floor effect). Similarly, controls may have no problem at all in attentional disengagement, thus exhibiting a large IOR effect for both cue types (i.e., a ceiling effect). Consequently, the results from Experiment 1 are not conclusive. We therefore conducted another experiment to clearly differentiate between the theory of attention disengagement and the theory of habituation/detection cost. In that experiment, we further examined whether patients with schizophrenia have deficits in attention disengagement or habituation/detection cost.

## Experiment 2

In Experiment 2, we used a similar single-cue procedure to that of Experiment 1. However, instead of manipulating the valence of the cue, we manipulated the valence of the target. We intended to explore empirically whether the supposed increased ability of threatening faces to capture attention might overcome or cancel out the cost of attention being captured again at the cue location (i.e., the IOR effect). Theoretically, this new experiment could help us to select a model to interpret the attentional deficit observed in patients. The attention disengagement view predicts no effect of target emotionality on IOR since everything is the same for all trials until the target is presented. By contrast, according to the habituation/detection cost theory, we should expect an effect of the emotionality of the target on IOR, in line with previous findings (39): when the target includes threatening information, a reduced or absent IOR should be observed, particularly in patients, as one should expect reduced habituation (and/or enhanced attentional capture) to threatening information (47).

### Participants

Two new groups of participants were recruited in this experiment (Table 3). The patient group was composed of 20 stable patients with schizophrenia from the Tianjing Psychiatry Hospital, Tianjing, China. As in Experiment 1, patients’ diagnoses were confirmed based on structured clinical interviews conducted in accordance with the fourth edition of the DSM-IV (53). Before the experiment took place, patients were carefully screened to rule out any potential disorders that might alter brain functioning (e.g., mental retardation). In addition, each patient was evaluated by at least two experienced psychiatrists in the hospital. The exclusion criteria were the same as in Experiment 1. The control group consisted of 15 undergraduate or graduate students recruited from Peking University, Beijing. All participants reported normal or correct-to-normal vision and being right-handed, and all were naïve as to the purpose of the experiment. Everything else was the same as in Experiment 1.

**Table 3:** **Characteristics of the groups studied in Experiment 2 (patients with schizophrenia and control participants)**.

Participants’ characteristics	Schizophrenia (*n* = 20)	Healthy controls (*n* = 15)	*t* Value	*p* Value
Age (years)	33.6 (1.77)	23.3 (0.733)	5.40	0.001
Gender (female ratio)	10/20	10/15	0.98	0.336
Education level (years)	8.50 (0.387)	13.27 (0.267)	9.45	0.001
Right-handedness	20/20	15/15		
Length of illness (years)	12.02 (1.59)			

*Standard error (SE) is shown in brackets for age, education level, and length of illness*.

### Apparatus and procedure

The stimuli and general procedure used were the same as in Experiment 1, except that the two types of faces (i.e., threatening face vs. scrambled face, luminance matched) were used as the target stimuli and the small square was used as the cue.

### Results and discussion

**Figure 3:**
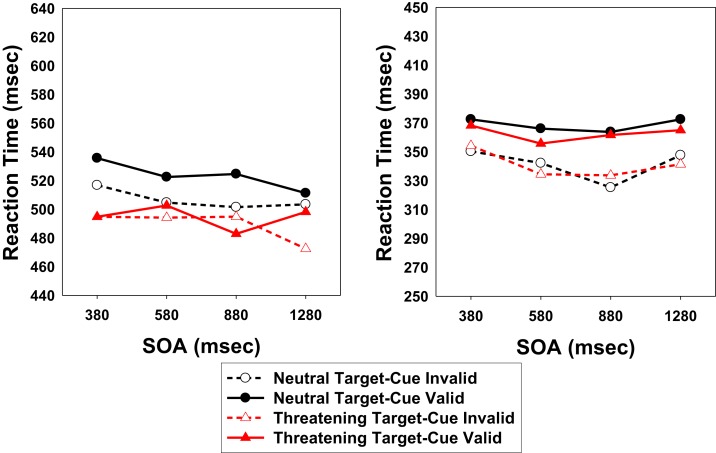
**Target detection times of Experiment 2, broken down by Cueing (cued vs. uncued location), Target valence (threatening vs. neutral), and Stimulus Onset Asynchrony (SOA)**. Left panel: patient group; right panel: control group.

As in Experiment 1, RTs above and below ±2.5 SD from the condition-specific mean were eliminated from the data analyses. Less than 5% of the trials were discarded following this criterion (controls: 4.5%; patients: 4.1%). Figure 3 presents the mean target detection times, broken down by SOA, Cueing, and Target Valence. In addition, Table 4 shows mean RTs and percentage of errors (misses). Correct RTs were subjected to a 2 × 4 × 2 × 2 mixed analysis of variance (ANOVA) with group (patients vs. controls) as the between-participants factor and SOA (380, 580, 880, and 1280 ms), Target Valence (threatening face vs. scrambled face), and Cueing (valid vs. invalid) as within-participants factors.

**Table 4:** **Mean reaction times (ms) and accuracy as a function of target valence (threatening vs. neutral), Cueing (valid vs. invalid), and SOA in Experiment 2**.

**EXPERIMENT 2**								
	SOA							
	380		580		880		1280	
	Cue_invalid	Cue_valid	Cue_invalid	Cue_valid	Cue_invalid	Cue_valid	Cue_invalid	Cue_valid
**RT (ms)**
Control neutral target	350.27 (9.93)	372.43 (8.78)	342.17 (15.61)	366.02 (13.21)	325.28 (11.17)	363.71 (13.88)	347.65 (10.52)	372.43 (11.51)
Control threatening target	354.18 (12.09)	368.21 (8.90)	334.41 (14.82)	355.73 (11.67)	333.68 (13.35)	361.62 (11.98)	341.37 (12.40)	364.96 (11.89)
Patient neutral target	516.78 (20.81)	535.63 (23.24)	504.63 (21.38)	522.48 (22.58)	501.49 (24.32)	524.58 (24.28)	503.41 (24.20)	511.27 (22.47)
Patient threatening target	494.74 (20.42)	494.78 (21.48)	494.17 (25.41)	502.62 (22.95)	494.84 (23.55)	482.89 (20.81)	472.51 (21.41)	498.14 (23.15)
**Accuracy**
Control neutral target	1.00 (0.00)	1.00 (0.00)	1.00 (0.00)	1.00 (0.00)	1.00 (0.00)	1.00 (0.00)	1.00 (0.00)	1.00 (0.00)
Control threatening target	1.00 (0.00)	1.00 (0.00)	1.00 (0.00)	1.00 (0.00)	1.00 (0.00)	1.00 (0.00)	1.00 (0.00)	1.00 (0.00)
Patient neutral target	0.96 (0.01)	0.95 (0.02)	0.96 (0.02)	0.98 (0.01)	0.97 (0.02)	0.95 (0.02)	0.97 (0.02)	0.96 (0.02)
Patient threatening target	0.97 (0.01)	0.97 (0.01)	0.94 (0.02)	0.98 (0.01)	0.97 (0.01)	0.98 (0.01)	0.96 (0.01)	0.96 (0.02)

*Standard error (SE) is shown in brackets*.

The analysis revealed a main effect of Cueing, *F*(1, 33) = 22.58, *p* < 0.001, a main effect of Group, *F*(1, 33) = 31.67, *p* < 0.001, and moderate evidence for the expected Cueing × Group interaction, *F*(1, 33) = 3.12, *p* = 0.087^2^. The main effect of Target Valence was significant, *F*(1, 33) = 24.89, *p* < 0.001. Importantly, the interaction between Target Valence and Group also reached significance, *F*(1, 33) = 14.21, *p* = 0.001, indicating that patients showed a much larger effect of target emotionality. A further analysis indicated that controls showed a clear IOR effect with both target valences [scrambled face, *F*(1, 16) = 20.64, *p* < 0.001; threatening face, *F*(1, 16) = 11.84, *p* = 0.003], although the effect was numerically smaller for threatening faces (scrambled face: 26 ms; threatening face: 20 ms). By contrast, patients only showed an IOR effect when the target was a neutral scrambled face (17 ms), *F*(1, 19) = 10.34, *p* = 0.005, and not when it was a threatening face (5 ms), *F*(1, 19) = 0.66, *p* = 0.425^3^. Specifically, across the three shortest SOAs, a clear IOR effect was observed for neutral expression faces (15 ms) but no IOR effect was observed for threatening faces (−1 ms). No other significant effects were found. Complete statistical tests are presented in the Appendix. As in Experiment 1, the analysis of errors (misses) only revealed a main effect of Group, indicating that patients missed the target (3.65%) more frequently than controls (0.18%), *F*(1, 33) = 6.17, *p* = 0.018.

In the patient group, RTs were much faster in Experiment 2 (mean: 503.4 ms) than in Experiment 1 (mean: 560.7 ms). This is consistent with the findings of Bourke et al. (52), according to which patients with schizophrenia show increased vigilance and selective attention to negative information. However, it is important to note that the attention disengagement view predicts no effect of target emotionality on IOR since everything is the same for all trials until the target is presented. Clearly, the attentional capture by the cue and the subsequent disengagement of attention from it should have been the same for neutral and emotional target trials. Therefore, the different data pattern observed in this experiment for threatening vs. neutral targets cannot be explained by different attentional disengagement for each target type. Below we discuss further the theoretical implications of this finding in terms of reduced habituation of attentional capture by emotionally relevant stimuli.

## General Discussion

In this research, we explored attention deficits in patients with schizophrenia using a standard cue–target covert orienting paradigm suitable to measure IOR. We also tested whether IOR might be better explained by the attention disengagement or the habituation/detection cost theories by manipulating the emotionality of the attention capturing events (i.e., cue and target). This helped us to understand the observed differences between patients and controls regarding the IOR effect. As noted, a key aspect of the current work was the manipulation of the cue and the target valence separately in different experiments.

In Experiment 1, we manipulated the emotional valence of the cues. The absence of an effect of emotionality of the cue over the cueing effect seems to be at odds with the traditional attention disengagement hypothesis, as no effect of emotionality was found in controls or patients. In particular, IOR was fully absent or delayed in patients similarly for neutral and threatening cues. If patients had a problem of attention disengagement, as suggested by Mushquash et al. (18), they should have a greater problem with threatening cues, from which it is supposedly more difficult to disengage attention (38). However, this was not the case in our study.

In Experiment 2, by contrast, we manipulated the emotional valence of the target. Controls exhibited IOR with both target types – although somehow small for the threatening target – by contrast, patients only exhibited IOR in neutral target trials (17 ms), not in threatening target trials (5 ms, non-significant effect). It is important to note that, according to the attention disengagement view of IOR (15, 18), when the cue has no emotional valence, the potential attention disengagement deficit observed in patients should be the same for either type of targets (i.e., the threatening face and the corresponding scrambled face). Specifically, trials with neutral and threatening targets were exactly the same before the target was presented, hence the nature of the target could not be predicted in advance. Therefore, attention should have been captured and disengaged from or maintained at the cued location equally in neutral and threatening target trials. Consequently, the differences in IOR can only be attributed to a different effectiveness of the two target types regarding attentional capture and detection processes (33).

We conducted two additional ANOVAs to confirm that the valence effect was different between the two experiments for controls and patients, respectively. As expected, the two-way Valence × Experiment interaction was significant in both ANOVAs, [controls, *F*(1, 28) = 6.52, *p* = 0.016; patients, *F*(1, 38) = 21.48, *p* < 0.001]. This indicated that the effect of emotionality only appeared when the target valence was manipulated. The fact that the effect of emotionality (i.e., its modulation of the cueing effect) was observed in Experiment 2 but not in Experiment 1 supported the attention habituation/detection cost theory of IOR [(28, 32); for a similar conclusion, see Ref. (57, 58)] against the attention disengagement theory of Mushquash et al. (18). This conclusion is also consistent with the considerable number of studies currently showing that disengagement of attention is neither necessary nor sufficient for IOR to be observed (22–27).

Considering that IOR is due to attention habituation/detection cost rather than attention disengagement, why did patients with schizophrenia exhibit a smaller IOR effect without cue-back but a normal IOR effect with cue-back in some studies? As we noted in the Section “Introduction,” a handful of studies seemed to suggest to Mushquash et al. (18) that the smaller or delayed IOR of patients with schizophrenia evidenced a kind of deficit in endogenous or voluntary control of attention. However, this conclusion seems far from conclusive. For instance, it is true that both Posner et al. (9) and Daban et al. (59) reported that patients with schizophrenia failed to show IOR without cue-back [also see Ref. (19, 60)]. Yet, Larrison-Faucher et al. (7) used the cue-back procedure to ensure that attention was drawn away from the initially cued location, but patients still showed a delayed onset of IOR. Interestingly, Sapir et al. (61) doubted that attention disengagement could explain the IOR deficit in schizophrenia as in their study (Experiment 1) patients did not show impaired disengagement of attention in a similar orienting task (see p. 369). In conclusion, it seems that the cue-back procedure may have an effect other than disengaging attention from the cued location.

In fact, recent research suggests that the cue-back procedure interrupts cue–target integration processes, which leads to facilitation effects, especially when discrimination rather than detection tasks are used [(22); for a review, see Ref. (32)]. The cue–target integration process may be intact or even enhanced in schizophrenia, so that patients are less biased to novelty. To the extent that the facilitation effect due to cue–target integration is eliminated when a cue-back procedure is used, patients with schizophrenia should mainly exhibit IOR. In other words, the fact that some previous studies only found IOR in patients with schizophrenia when a cue-back experimental procedure was used does not necessarily mean that such patients have a deficit in endogenous or voluntary control of attention [for a similar view, see Ref. (61)].

In the present study, results for healthy controls (Experiment 1) replicated the pattern found by Stoyanova et al. (62) and Lange et al. (63): when the threatening cue appeared during the cue period, there was no effect of emotionality for healthy participants (i.e., a ceiling effect). However, when the threatening face appeared during the target period (Experiment 2), the modulation of emotionality over the observed cueing effect was expected to appear, with smaller IOR for threatening targets. Yet, we only observed a mild effect of emotionality in the control group. It should be noted that Baijal and Srinivasan (36) also observed only a small reduction in the IOR effect with sad compared to happy schematic faces in a detection task. We attribute this to the use of a simple detection task. In a recent study, for instance, Pérez-Dueñas et al. (39) observed the expected modulation or IOR in an emotion categorization task in which the target was a neutral or emotional face and participants were asked to categorize the faces as either neutral or emotional. In another study in which emotional words instead of faces were used as targets, the authors reported similar results only in participants who scored high in trait anxiety (64). It should be noted that in both studies participants were required to perform an emotional categorization task rather than simply detect the target as in our procedure. The use of an emotional categorization task is very likely to have enhanced emotional processing and therefore the effect of emotionality on IOR. In the present study, however, despite the use of a detection task, the IOR effect vanished completely in patients when threatening targets were used. This might indicate that emotionally negative targets are less prone to habituation. In other words, threatening faces may be particularly appropriate to capture attention and emotional categorization might emphasize the processing of emotion. Whether the IOR effect is eliminated or reduced in all participants [as in Ref. (39)] or in specific populations (e.g., high anxiety group or patients with schizophrenia) may depend on the task demands and the material/stimuli used.

We should add one caveat here. In the present research, patients and controls were not perfectly matched in education and age and we did not have the chance to measure patients’ intelligence. It will therefore be helpful for future studies to replicate the observed pattern of data while controlling for all possible differences between controls and patients. However, we believe that these factors did not contribute significantly to the differences observed between the two groups. First, we used a very simple detection task and neither of the groups had any problems performing it. Second, age differences between groups were not large and no IOR differences have previously been reported within such small age differences. If patients had general problems with the inhibitory mechanism, it would have affected both the neutral and emotional stimuli detection. The most important finding of our study was that patients had difficulties in habituating to previously attended information, thus not exhibiting IOR, particularly in the case of emotional targets.

In summary, the present study suggests that attentional deficits in schizophrenia may not be related to impaired attention disengagement. Instead, patients with schizophrenia exhibited a deficit in detecting new information at a previously cued location. From this perspective, such patients may have a deficit in detecting new information and considering it as new in the current context. Therefore, their attention deficits may be more related to defective novelty detection/habituation processes. Therefore, we believe that caution should be exercised when attempting to identify individuals with schizophrenia, especially when interpreting their attentional deficits. In addition, our study provides converging evidence to disentangle the two current different theoretical approaches to understand IOR. Such evidence supports the new approach proposed by Dukewich (28) and Lupiáñez (32), according to which the presence of a similar preceding event (i.e., the cue) leads to weaker attentional capture by new information and to a cost in detecting the appearance of new events at locations where attention was captured previously.

## Conflict of Interest Statement

The authors declare that the research was conducted in the absence of any commercial or financial relationships that could be construed as a potential conflict of interest.

## Footnotes

^1^Given the considerable difference in RTs between groups, we performed a proportional RT analysis. For each participant, the mean per condition was divided by the overall mean for that participant [for a similar approach, see Ref. (65)]. Taking into account between-group RT differences, this analysis showed a highly significant Group × Cueing interaction [*F*(1,33) = 9.40, *p* = 0.004].

^2^If proportional RT was used for the analysis instead, this result was significant [*F*(1,33) = 6.72, *p* = 0.014].

^3^Note that the Target Valence × Cueing interaction approached significance, *F*(1,33) = 3.11, *p* = 0.087. Both groups tended to show a smaller IOR for threatening face targets, although the effect was clearer in patients, who did not exhibit IOR for threatening face targets.

## Appendix

### ANOVA values for each experiment.

#### Experiment 1 – DV: mean RT (*p ≤ 0.05; **p ≤ 0.001).

**Table:**

*N* = 35	df	*F*	*p* Value
SOA	(2, 72)	8.25	0.000**
Cueing	(1, 33)	18.59	0.000**
Cue Valence	(1, 33)	0.80	0.377
Group	(1, 33)	41.26	0.000**
SOA*Cueing	(3, 99)	1.09	0.358
SOA*Cue Valence	(2, 78)	0.17	0.878
SOA*Group	(3, 99)	4.47	0.005**
Cueing*Cue Valence	(1, 33)	0.00	0.991
Cueing*Group	(1, 33)	3.67	0.064
Cue Valence*Group	(1, 33)	0.01	0.915
SOA*Cueing*Cue Valence	(3, 99)	2.06	0.110
SOA*Cueing*Group	(3, 99)	0.49	0.687
SOA*Cue Valence*Group	(3, 99)	0.33	0.807
Cueing*Cue Valence*Group	(1, 33)	0.27	0.608
SOA*Cueing*Cue Valence*Group	(3, 99)	1.87	0.140

#### Experiment 1 – DV: proportional RT (*p ≤ 0.05; **p ≤ 0.001).

**Table:**

*N* = 35	df	*F*	*p* Value
SOA	(2, 68)	10.72	0.000**
Cueing	(1, 33)	33.48	0.000**
Cue Valence	(1, 33)	1.96	0.171
SOA*Cueing	(3, 99)	1.58	0.199
SOA*Cue Valence	(3, 99)	0.13	0.941
SOA*Group	(3, 99)	5.35	0.002**
Cueing*Cue Valence	(1, 33)	0.02	0.897
Cueing*Group	(1, 33)	9.40	0.004**
Cue Valence*Group	(1, 33)	0.07	0.795
SOA*Cueing*Cue Valence	(3, 99)	1.51	0.216
SOA*Cueing*Group	(3, 99)	0.69	0.562
SOA*Cue Valence*Group	(3, 99)	0.50	0.684
Cueing*Cue Valence*Group	(1, 33)	0.34	0.565
SOA*Cueing*Cue Valence*Group	(3, 99)	1.37	0.257

#### Experiment 2 – DV: mean RT (*p ≤ 0.05; **p ≤ 0.001).

**Table:**

*N* = 35	df	*F*	*p* Value
SOA	(2, 74)	3.18	0.042*
Cueing	(1, 33)	22.58	0.000**
Target Valence	(1, 33)	24.89	0.000**
Group	(1, 33)	31.67	0.000**
SOA*Cueing	(3, 99)	0.34	0.799
SOA*Target Valence	(3, 99)	0.34	0.796
SOA*Group	(3, 99)	1.61	0.191
Cueing*Target Valence	(1, 33)	3.11	0.087
Cueing*Group	(1, 33)	3.12	0.087
Target Valence*Group	(1, 33)	14.21	0.001**
SOA*Cueing*Target Valence	(3, 99)	2.52	0.063
SOA*Cueing*Group	(3, 99)	0.90	0.445
SOA*Target Valence*Group	(3, 99)	2.02	0.116
Cueing*Target Valence*Group	(1, 33)	0.36	0.551
SOA*Cueing*Target Valence*Group	(3, 99)	1.21	0.312

#### Experiment 2 – DV: Proportional RT (*p ≤ 0.05; **p ≤ 0.001).

**Table:**

*N* = 35	df	*F*	*p* Value
SOA	(2, 72)	4.57	0.011*
Cueing	(1, 33)	24.55	0.000**
Target Valence	(1, 33)	31.83	0.000**
SOA*Cueing	(3, 99)	0.48	0.695
SOA*Target Valence	(3, 99)	0.56	0.641
SOA*Group	(3, 99)	2.11	0.104
Cueing*Target Valence	(1, 33)	2.92	0.097
Cueing*Group	(1, 33)	6.72	0.014*
Target Valence*Group	(1, 33)	13.97	0.001**
SOA*Cueing*Target Valence	(3, 99)	2.28	0.084
SOA*Cueing*Group	(3, 99)	1.10	0.352
SOA*Target Valence*Group	(3, 99)	2.04	0.113
Cueing*Target Valence*Group	(1, 33)	0.12	0.728
SOA*Cueing*Target Valence*Group	(3, 99)	0.81	0.492
